# Environmental and Sanitary Conditions of Guanabara Bay, Rio de Janeiro

**DOI:** 10.3389/fmicb.2015.01232

**Published:** 2015-11-20

**Authors:** Giovana O. Fistarol, Felipe H. Coutinho, Ana Paula B. Moreira, Tainá Venas, Alba Cánovas, Sérgio E. M. de Paula, Ricardo Coutinho, Rodrigo L. de Moura, Jean Louis Valentin, Denise R. Tenenbaum, Rodolfo Paranhos, Rogério de A. B. do Valle, Ana Carolina P. Vicente, Gilberto M. Amado Filho, Renato Crespo Pereira, Ricardo Kruger, Carlos E. Rezende, Cristiane C. Thompson, Paulo S. Salomon, Fabiano L. Thompson

**Affiliations:** ^1^Institute of Biology, Federal University of Rio de JaneiroRio de Janeiro, Brazil; ^2^Laboratório de Sistemas Avançados de Gestão da Produção, COPPE, Federal University of Rio de JaneiroRio de Janeiro, Brazil; ^3^Centre for Molecular and Biomolecular Informatics, Radboud Institute for Molecular Life Sciences, Radboud University Medical CentreNijmegen, Netherlands; ^4^Instituto de Estudos do Mar Almirante Paulo MoreiraRio de Janeiro, Brazil; ^5^Oswaldo Cruz Institute, Fundação Oswaldo CruzRio de Janeiro, Brazil; ^6^Instituto de Pesquisas Jardim Botanico do Rio de JaneiroRio de Janeiro, Brazil; ^7^Laboratory of Marine Chemical Ecology, Insitute of Biology, Federal Fluminense UniversityNiteroi, Brazil; ^8^Laboratory of Enzimology, Institute of Biology, University of BrasíliaBrasília, Brazil; ^9^Laboratory of Environmental Sciences (LCA-UENF), CamposBrazil

**Keywords:** Guanabara Bay, anthropogenic impacts, sanitary conditions, bacteria, microalgae, pollution

## Abstract

Guanabara Bay is the second largest bay in the coast of Brazil, with an area of 384 km^2^. In its surroundings live circa 16 million inhabitants, out of which 6 million live in Rio de Janeiro city, one of the largest cities of the country, and the host of the 2016 Olympic Games. Anthropogenic interference in Guanabara Bay area started early in the XVI century, but environmental impacts escalated from 1930, when this region underwent an industrialization process. Herein we present an overview of the current environmental and sanitary conditions of Guanabara Bay, a consequence of all these decades of impacts. We will focus on microbial communities, how they may affect higher trophic levels of the aquatic community and also human health. The anthropogenic impacts in the bay are flagged by heavy eutrophication and by the emergence of pathogenic microorganisms that are either carried by domestic and/or hospital waste (e.g., virus, KPC-producing bacteria, and fecal coliforms), or that proliferate in such conditions (e.g., vibrios). Antibiotic resistance genes are commonly found in metagenomes of Guanabara Bay planktonic microorganisms. Furthermore, eutrophication results in recurrent algal blooms, with signs of a shift toward flagellated, mixotrophic groups, including several potentially harmful species. A recent large-scale fish kill episode, and a long trend decrease in fish stocks also reflects the bay’s degraded water quality. Although pollution of Guanabara Bay is not a recent problem, the hosting of the 2016 Olympic Games propelled the government to launch a series of plans to restore the bay’s water quality. If all plans are fully implemented, the restoration of Guanabara Bay and its shores may be one of the best legacies of the Olympic Games in Rio de Janeiro.

## Introduction: General View of the Issue and Historical Background

**FIGURE 1:**
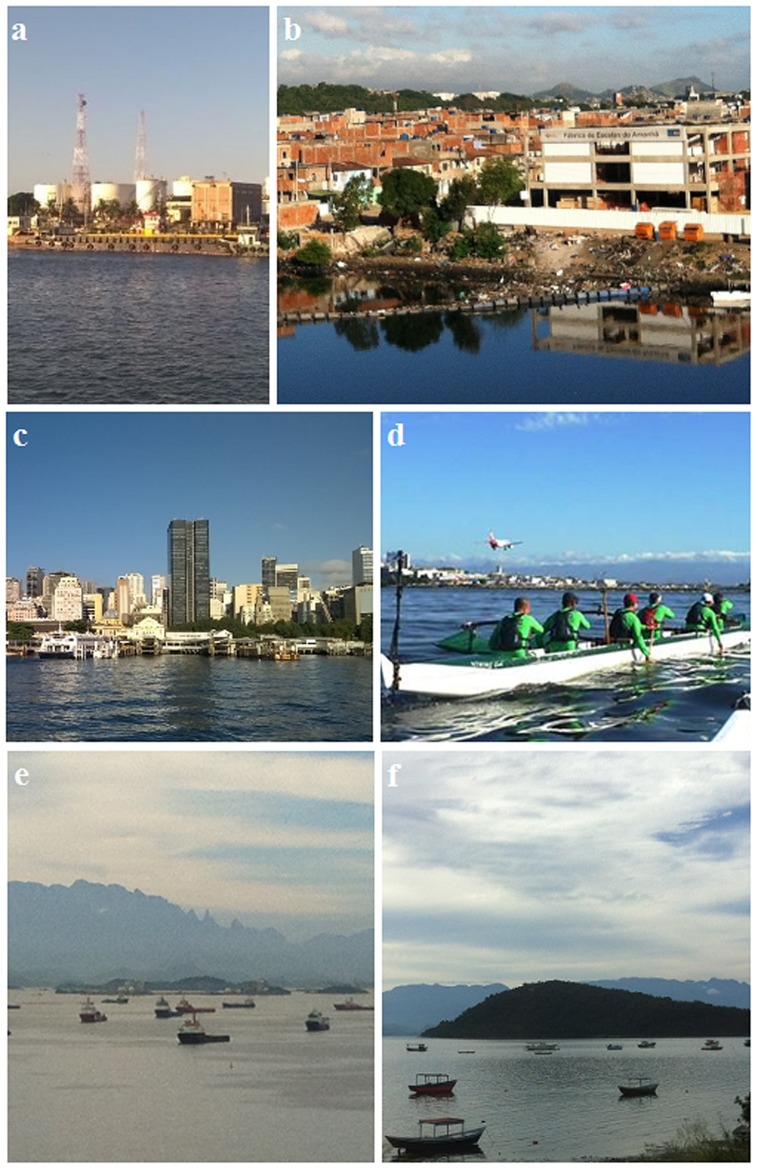
**Composition of pictures showing degraded (a,b) and non-degraded areas of Guanabara Bay (f), which still have some fringing mangrove system.** There are different urban landscapes surrounding the bay, such as industries **(a)**, slums **(b)**, metropolis **(c,d)**. The bay has social-economic importance and it is used, among others, as harbor **(e)**, for artisanal fisheries **(f)**, and recreational purposes **(d)**. Pictures by: **(a)** and **(c)**: Michelle Vils; **(d)**: Wanderson F. de Carvalho; **(b, e)** and **(f)**: Giovana O. Fistarol.

Guanabara Bay is the second largest bay on the coast of Brazil, and is situated along the northeast coast of Rio de Janeiro city, one of the most populated urban areas of the world^1^ (26^th^, according to Demographia, 2014), and one of the most important cities in Brazil. Rio de Janeiro is world-widely known for its many natural beauties, among which are included Guanabara Bay. This environment harbored a great diversity of organisms with undeniable ecological importance, and has been an important landmark of Brazil, being one of the first settlement sites, and, from the beginning, one of country’s most important economic regions. The importance of the bay, however, did not prevent the occurrence of a series of environmental impacts that are currently reflected on its present state (**Figure 1**).

**FIGURE 2:**
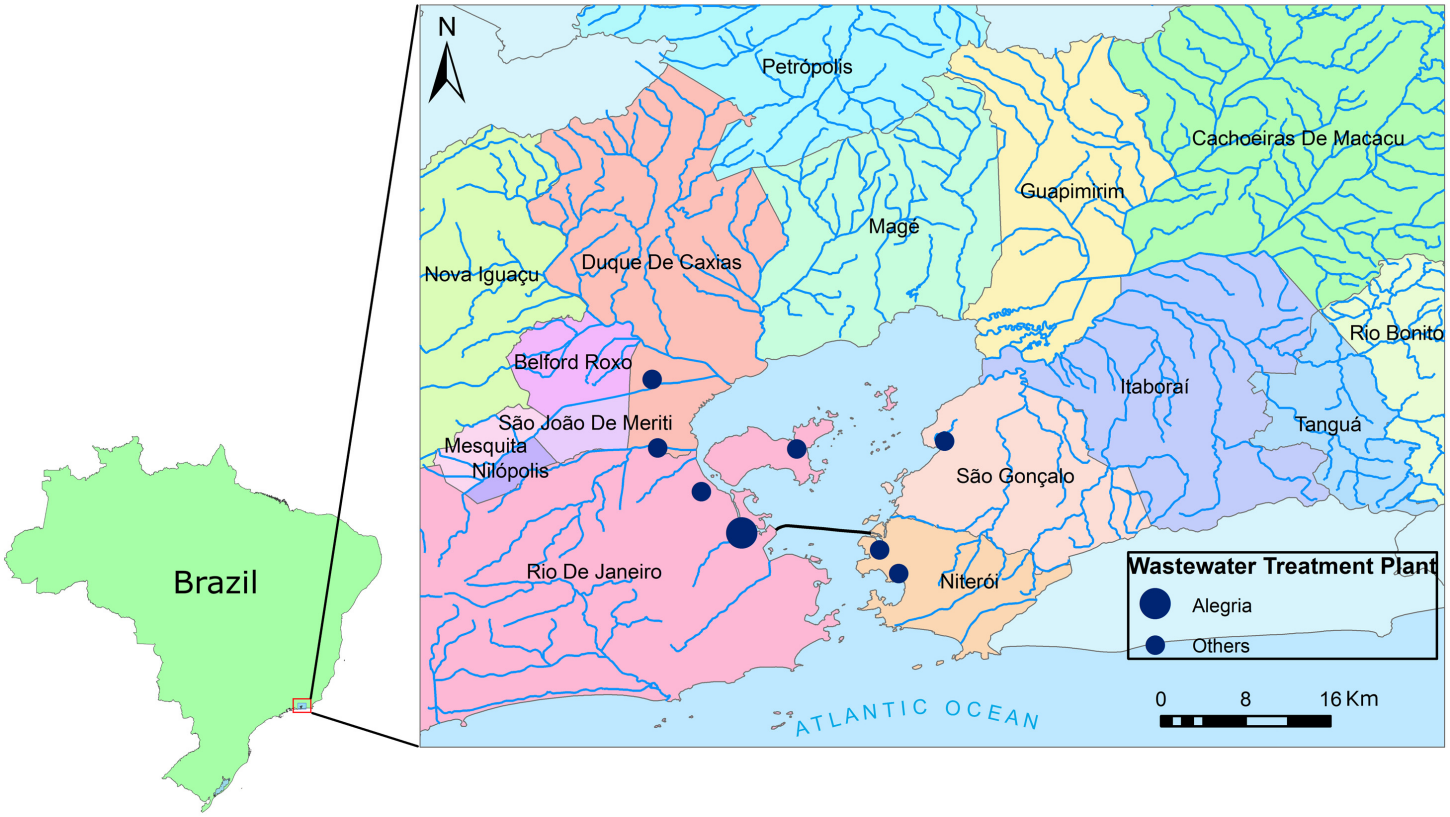
**Location of Guanabara Bay, Southeastern Brazil.** The cities around the bay and the rivers that form the bay’s drainage area are shown, as well as the location of sewage wastewater treatment plants (WWTPs) (WWTP location obtained from Comitê de Bacia da Baía de Guanabara, SIG-RHGB, http://www.comitebaiadeguanabara.org.br/sig-rhbg/).

Guanabara Bay drainage basin covers 4081 km^2^, being, at the same time an important water resource, and the receptor of most of the liquid eﬄuents produced along its drainage basin, which covers totally or partially 16 municipalities (Belford Roxo, Cachoeira de Macacu, Duque de Caxias, Guapimirim, Itaboraí, Magé, Mesquita, Nilópolis, Niterói, Nova Iguaçu, Petrópolis, Rio Bonito, Rio de Janeiro, São Gonçalo, São João de Meriti, and Tanguá) (SEMADS, 2001). From these, the largest and most important is Rio de Janeiro (**Figure 2**).

The current environmental and sanitary conditions of Guanabara Bay results from the cumulative impacts and environmental changes that occurred since the first European settlers got established in the XVI Century. Since then, the region went through a series of economic cycles that started with timber exploitation (especially *Caesalpinia echinata*, known as Pau-Brasil), sugar-cane cultivation, mining, and then cultivation of coffee beans and orange. The impacts started to escalate around 1930, when industrialization of the area began, also bringing urbanization and a sharp population increase (Amador, 1997). Population density is currently high, with ca. 11.8 million inhabitants in the Rio de Janeiro City metropolitan area alone^2^ (IBGE, 2010). Moreover, this is one of the most industrialized coastal areas of Brazil, harboring more than 16000 industries, gas and oil terminals and two ports^2^ (IBGE, 1985; Coelho, 2007). On the other hand, sewage and water treatment is still very limited. Both industrial and urban contaminants are discharged into the bay or in the sea (disposal systems in Ipanema Beach and Barra da Tijuca). Other anthropogenic impacts include destruction of habitats, poor fisheries management, sedimentation, flooding, landslides, and various public health problems.

Attention toward Guanabara Bay pollution increased together with the increase in environmental awareness around the 1990’s^3^ (Rio de Janeiro hosted the UN conference on environment and development, ECO 92, in 1992, which produced the Agenda 21), triggering some governmental efforts to recover the bay’s water quality. One of the largest actions was the Program for Remediation of Guanabara Bay (PDBG) that began in 1994, as a cooperation between the Inter-American Development Bank, the government of Rio de Janeiro State, and the Japan Bank for International Cooperation (JBIC). This program planned to implement a large set of sewage treatment plants at strategic locations within the bay’s drainage basin. However, several plants were not concluded and others are not fully functional (e.g., some plants are still not connected to sewage collection and disposal systems) (Coelho, 2007). Currently, efforts toward the recovery of Guanabara Bay have gained new inputs, motivated by the fact that the city of Rio de Janeiro is going to host the 2016 Olympic Games. As Guanabara Bay will be the venue for various outdoor aquatic sports during the games, the government had to commit with a series of measures to ensure safe water quality levels for the athletes.

In this article we present an overview of the environmental and sanitary conditions of Guanabara Bay, and its consequences to marine life and human health. We address how the impacts have affected the bay, and then focus on the potentially pathogenic and toxic microorganisms, and on the possible implication of these organisms to higher trophic levels. We summarize the current efforts being made to restore water quality of the bay, and show the importance of implementing a continuous monitoring program to produce consistent datasets and to follow up restoration initiatives. Through the examination of available information about the impacts suffered and by comparing it to what has happened in other coastal environments around the world, we provide an assessment about the understanding of the system, and the information gaps that require further research.

## Guanabara Bay Hydrography

Guanabara Bay is part of a large ecosystem that forms the Guanabara Bay drainage basin. With 4081 km^2^, the basin is drained by 50 rivers and streams (SEMADS, 2001), six of which are responsible for 85% of the 100 m^3^ s^-1^ total mean annual freshwater discharge into the bay: Guapimirim (20.8%), Iguaçu (16.7%), Caceribu (13.7%), Estrela (12.7%), Meriti (12.3%), and Sarapuí (9.3%) (Coelho, 2007). The freshwater discharge ranges from 33 m^3^ s^-1^ in the dry austral winter to 186 m^3^ s^-1^ in the rainy austral summer (Kjerfve et al., 1997). Sedimentation rates are high, varying from 0.6 cm year^-1^ near the mouth to 4.5 cm year^-1^ in the inner part of the bay, mostly as a result of deforestation of the drainage basin and channelization of rivers (Amador, 1980). Besides, the surface area of the bay has been reduced by 10% as a result of land reclamation for construction of two commercial airports, roads, bridges, and residential areas (Amador, 1980; Coelho, 2007). Nevertheless, there are still circa 90 km^2^ of a fringing mangrove system bordering the inner margins of the bay, which includes the Guapimirim Environmental Protection Area (Pires, 1992). Land-use around the bay changed, with the urban area increasing in 80 km^2^, pastures increasing in 150 km^2^, and forests decreasing in nearly 100 km^2^ (Coelho, 2007). According to the map of land-use from the Guanabara Bay Watershed Committee^4^ (Comite de Bacia da Baía de Guanabara) the main land uses on the watershed are high and medium density urban areas, pasture, and forest (this last one being found mostly on the mountain areas), then there are some areas used for agriculture and some fringing mangrove system. The proportion of urban and rural population around the bay ranges from a maximum of 34.7% of rural population to 100% of urban population; most municipalities, in fact, have zero to ca. 5% of rural population^2^ (IBGE, 2000). Furthermore, a significant pattern is the uncontrolled and disordered occupation of land by slums on the hills in Rio de Janeiro metropolitan area, taking over the remaining forest areas. These vast areas lack sewage collection and disposal systems.

The bay has a surface of 381 km^2^, with 22 islands (the largest one being Governador Island, with 40.8 km^2^) (SEMADS, 2001). It has a narrow and relatively deep entrance of 1.6 km, and measures approximately 30 km in its west to east axis, and 28 km in its north to south axis, with a mean water volume of 1.87 billion m^3^. Most of the bay (84%) has <10 m depth, with a maximum depth of 58 m on its central channel (Ruellan, 1944). From the bay’s mouth to ca. 7 km inward, it has a sandy bottom that extends from the adjacent continental shelf, with some isolated sand areas at northeast and southwest of Governador Island. Otherwise, the bottom of the bay consists mostly of mud deposits (Amador, 1980).

Like in other coastal bays (e.g., Chesapeake Bay; Shi et al., 2013), the tidal regime is an important component of Guanabara Bay’s water circulation (Mayr et al., 1989; Paranhos et al., 1998). Tide in the bay is mixed, but mainly semidiurnal, with a mean tidal range of circa 0.7 m (spring tidal range: 1.1 m, neap tidal range: 0.3 m), without significant spatial variance. The associated peak tidal ebb and flood volume fluxes with the ocean are in the order of 16 × 10^3^ m^3^ s^-1^. Currents are also semi-diurnal, with flood currents (∼1.25 m s^-1^ at the surface and ∼1 m s^-1^ near the bottom) being faster than ebb currents (∼1 m s^-1^ at the surface and ∼0.55 m s^-1^ near the bottom). Currents also intensify at the entrance of the bay and between the mainland and the Governador Island, because of a narrowing of the channel in these areas. Circulation inside the bay is a composition of gravitational (which presents bidirectional flux: toward the ocean in the surface, and toward the continent near the bottom) and residual tide circulation, which are affected by the prevailing wind. As a result, it takes 11.4 days to renew 50% of the water in the Bay (Kjerfve et al., 1997). This relatively short residence time is one of the main factors explaining why water quality is not worse, considering the amount of untreated sewage discharged into the bay. However, it is important to have in mind that this renewal is not the same in all parts of the bay. In fact the innermost regions of the bay, which are the ones receiving most urban sewage, have lower circulation and a longer residence time, causing accumulation of organic matter and other contaminants, making these the most polluted areas of the bay. The stronger effects of tidal currents near the mouth of coastal bays compared to inner parts of the water bodies is well known. In Chesapeake Bay, water-quality parameters such as total suspended solids and chlorophyll-*a* (Chla) concentration decrease in the lower bay region under high tidal currents, while the tidal effect are small or negligible in the middle and large part of the upper bay region (Shi et al., 2013). Likewise, for Guanabara Bay it was found significant differences in the water quality between tidal stages (the quality being better at high tide, at the maximum tide dilution). It was also found that the influence of tidal stage was twice as high as the influence of rainy/dry season, and the influence of tides on the variation of several parameters, such as salinity, nutrients, Chla, and fecal coliforms concentration (Paranhos et al., 1998).

Salinity in the bay ranges from 13 to 36 (Mayr et al., 1989). However, a decadal trend of decreasing salinity has been detected, especially in the inner parts of the bay that are under high anthropogenic influence (Paranhos et al., 1993). Due to coastal versus riverine contributions to bay waters, vertical gradients can be up to 20°C and 18 salinity units (Paranhos et al., 1998). Vertical stratification of the water column is most pronounced in the shallow inner parts of bay, near stream discharges, and is correlated to the rainy and dry seasons: during the rainy period (October to April) temperatures are higher and salinities are lower, with the presence of thermo- and haloclines, while during the dry season (May to September) temperatures are lower, salinities are higher and there is no stratification (Paranhos and Mayr, 1993; Paranhos et al., 1993). Interestingly, the deep central channel traps cold salty water (21.1°C with a salinity of 34.4 at 24 m, see **Table 1**) (Kjerfve et al., 1997). This hydrographic heterogeneity between different parts of the bay shows that it cannot be considered as a homogeneous environment, and sub-regional dissimilarities should always be considered in its assessments and monitoring.

**Table 1:** Differences in water quality for different areas of Guanabara Bay, and other significant parameters: **(A)** near the entrance of the Bay, **(B)** station located between Ilha do Governador and Ilha do Fundão, on the west part of the Bay, close to the continent; **(C)** at the northwest part, close to the discharge of Rivers Iguaçu and Sarapui; **(D)** at the central channel at 24 m deep (see also **Figure 2** for stations location).

Area	Total N (μM)	Total P (μM)	Chla mg m^-3^	Vibrio	OD (mg l^-1^)	Fecal coliform (MPN 10^3^ 100ml^-1^)	Salinity	T°C	Suspended solids
A	0.6–68.3	0.05–7.4	0–58.2	171,53 ± 164,19		0.206 ± 0.17^∗^	32	22	
B	5–346.3	0.2–26.4	7.2–483.5	4508,15 ± 4825,66	0.28–3.97 (ml l^-1^)	132.53 ± 239.8^∗^		26	>25 mg l^-1^
C			> 125			41.08 ± 56^∗^	21	25	
D	50.9 ± 26.3 (s) 50.9 ± 18 (b)	2.7 ± 1 (s) 2.5 ± 1 (b)	19 ± 15.6	199,27 ± 262,68	8.76 (ml l^-1^)	1.05 ± 3.32^∗^	34.4	21.1	

*^∗^Annual mean calculated from data by INEA. When data was available, differences are shown for surface (s) and bottom water (b), and presented as mean ± SD. Data were retrieved from several sources: JICA (1994), Kjerfve et al. (1997), Paranhos et al. (1998), Paranhos et al. (2001), Marques et al. (2004), Gregoracci et al. (2012), INEA (2013; 2014a).*

## Water Quality: Pollutants in the Bay

### Types of Pollutants

Eutrophication of coastal waters, due to human activities, is a world-wide problem. It has been estimated that the export of P to the oceans has increased threefold, and N had even a higher increase in the last four decades (e.g., N increased more than 10-fold into the rivers entering the North Sea, and by six to eightfold in coastal waters of the northeastern United States generally and to Chesapeake Bay specifically) (Boynton et al., 1995; Howarth, 1998; Smil, 2001; Anderson et al., 2002). In Guanabara Bay there are several sources of contaminant’s discharge into the bay, from untreated domestic eﬄuents to industrial waste, which causes inputs of organic matter, nutrients, hydrocarbons, heavy metals, and large amounts of suspended solids. Domestic waste is responsible for discharging organic matter, and potentially pathogenic microorganisms. An average of 50.4% (*SD* = 17.9%) of the urban households are connected with sewage treatment system in the municipalities within the basin. Rio de Janeiro city has 78% of the households connected with sewage system^2^ (IBGE, 2000). However, these data should be looked with caution, since it considers only “permanent” households, i.e., the households with a regular legal situation with the municipalities. It does not consider all the households in the slums, which houses a very large population living irregularly and marginally in terms of public services and often not included in the statistics. All the domestic sewage from households that are not connected with the sewage system are discharged directly, untreated, into the bay or in the rivers of the basin. As an example, approximately 14% of households have no bathrooms and tap water in the Duque de Caxias area (Comissão Nacional Sobre Determinantes Sociais da Saúde, 2008). The lack of sanitary conditions and sewage treatment system in the poorest areas around Guanabara Bay, e.g., Duque de Caxias area, is directly reflected on infant mortality, which reaches 23,9%, contrasting with the areas served by sewage system, where the infant mortality is 4% (Coelho, 2007; Comissão Nacional Sobre Determinantes Sociais da Saúde, 2008). According to Coelho (2007), there has always been a deficit between the produced and treated sewage. In 2005, this deficit was 13 m^3^s^-1^ (treated sewage discharge was 7 m^3^ s^-1^, while untreated was 20 m^3^ s^-1^). The most recent estimation is that 18 m^3^s^-1^ of untreated sewage is discharged into the bay, although the data is not yet in the scientific peer reviewed literature^5^. Coelho (2007) also draws attention to the problem of stormwater runoff, which has been overlooked by the authorities. Stormwater runoff is collected in the same systems that receive sewage, which are undersized to receive all these eﬄuents. According to official information from the State Government of Rio de Janeiro, the efforts being made toward increasing the capacity of wastewater treatment plants (WWTPs), including the construction of new plants, to reach the goals set before the Olympic Committee have increased the percentage of treated sewage from 17 to 49%^6^ (O Globo).

The estimated daily organic load into the bay is 470 t of biological oxygen demand (BOD rages from 1-24 mg l^-1^), and around 150 t of industrial wastewater, which comes from the almost 17,000 industries among pharmaceutical and refineries, besides oil and gas terminals and two ports^2^ (IBGE, 1985; Coelho, 2007). Furthermore, it is estimated that 18 t day^-1^ of petroleum hydrocarbons enter the bay, mostly from urban runoff (Wagener et al., 2012). The industries around the bay are responsible for 20% of the organic load input, and for most of the toxic substances discharged into the bay. It has been estimated that 10,000 t month^-1^ of hazardous substances are produced in the Guanabara Bay’s Basin (Coelho, 2007). Solid waste is also present and visible in several areas of the bay’s margins (**Figure 1b**), including beaches that are commonly used for recreation. The majority of the beaches within the bay are not appropriate for swimming. Coelho (2007) estimated that 813 t day^-1^ of solid waste reaches the bay via its eﬄuents. Solid waste affects fisheries, navigation, leisure, tourism, the native fauna, and the landscape’s aesthetical value. Furthermore, solid waste also results in slurry production, from which an unknown amounts leaches to the bay.

### Distribution of Pollutants in the Bay

The first indication of the pollution impacts in the bay is the low transparency of the water (as low as 0.7 m) (Mayr et al., 1989; Valentin et al., 1999). In general, the innermost sites are characterized by low salinity (due to fluvial and sewage input), very high nutrient concentrations, and often low levels of dissolved oxygen as a result of the eutrophication process. The outermost waters present lower nutrient concentrations and higher levels of salinity and dissolved oxygen. Between these two extremes, a gradient of decreasing pollution is created toward the Atlantic Ocean.

**FIGURE 3:**
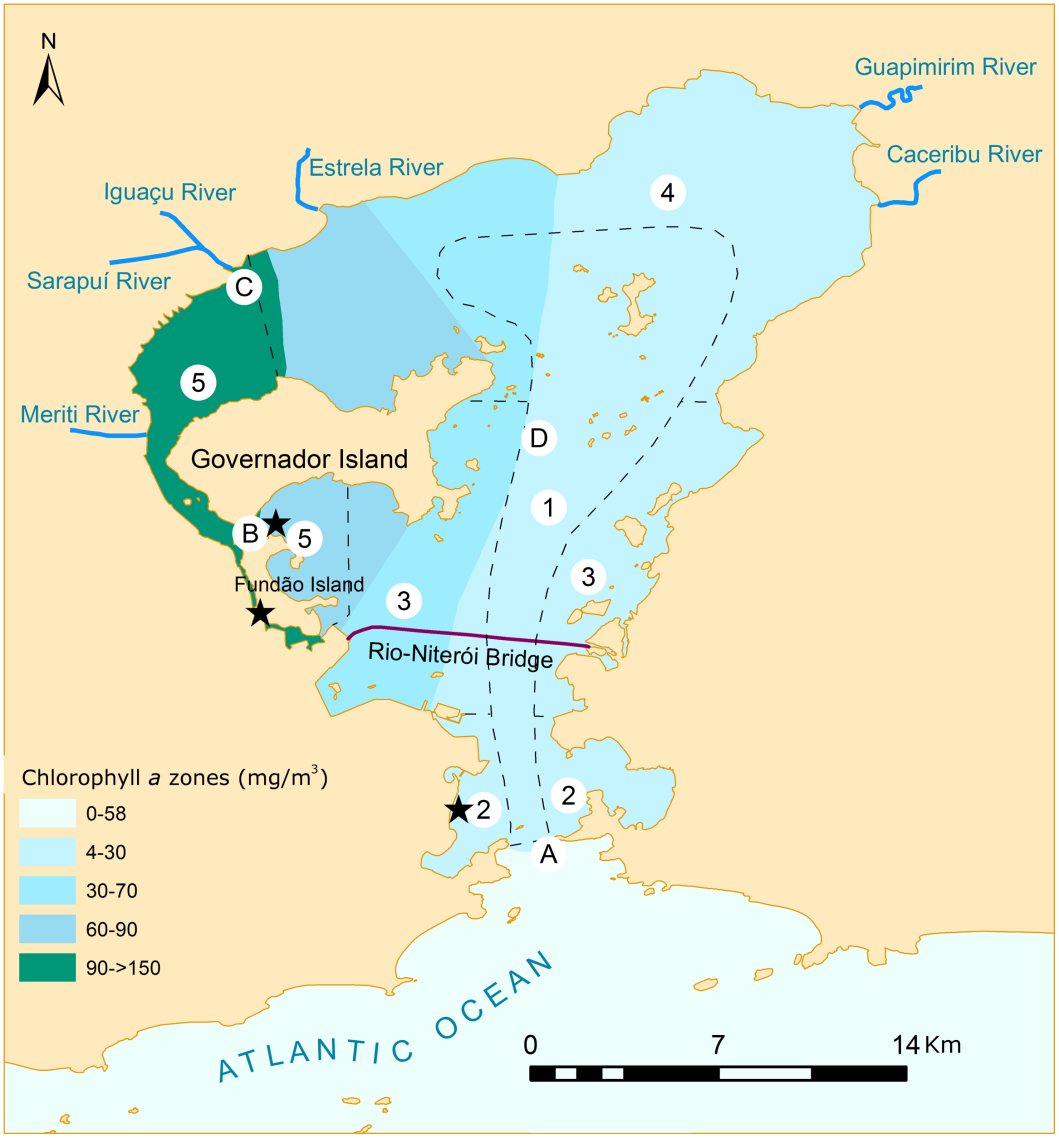
**Map of Guanabara Bay showing different parameters that indicate water quality throughout the sampling points (A–D) cited in the text.** Sites where resistant bacteria were found are marked with a star (★), according to Coutinho et al. (2014). The bay was divided in sections (1–5, dashed lines) of various water quality levels (according to Mayr et al., 1989): *section 1.* better water quality conditions; *section 2.* areas with high circulation, but subjected to high organic load; *section 3.* deteriorated areas under strong influence of urban and industrial contaminants; *section 4.* areas under the influence of less polluted rivers and that still maintain a fringe of mangrove; *section 5.* the most deteriorated areas with low circulation and high input of contaminants. Water quality zones show a good correspondence with chlorophyll *a* (Chla) concentrations recorded in the literature (Mayr et al., 1989; Paranhos et al., 1998; Paranhos et al., 2001; Santos et al., 2007) which are shown here by different colors.

The gradients of water quality in the bay are mainly controlled by: (i) seasonal changes between the rainy (September to May) and dry periods (June to August), which influence the hydrography of the bay, stormwater runoff, and also the discharge of untreated wastewater into the basin’s rivers; (ii) discharge of contaminants (influenced by the seasonal patterns, and a consequence of the population distributional patterns, and implementation or not of sewage treatments), and (iii) circulation and influence of marine water (which have greater influence on the south and southeast parts of the bay, and is mainly controlled by the tides) (Mayr et al., 1989; Paranhos et al., 1998; Valentin et al., 1999). Thus, the areas with the worst water quality are on the northern and northwestern parts of the bay, where two characteristics interact synergistically to aggravate the problem (it is the region receiving the greatest load of wastewater, and the one with the lowest water circulation). In the areas where dilution by seawater is higher as a consequence of tidal mixing (i.e., the central channel and the eastern part of the bay), water quality is significantly better (**Table 1** and **Figure 3**).

This pattern of water quality gradient (worse conditions on the N and NW parts, and better conditions on the E and on the central channel) is observed for most of the parameters. Values of total mean nitrogen ranged from 0.6 to 68.3 μM close to the entrance of the bay (Area A, on **Table 1**, for location see **Figure 3**), to 5–346 μM between Governador Island and Fundão Island (Area B, on **Table 1**), on the west part of the bay, close to the mainland. Total phosphorus ranged from 0.05 to 7.4 μM on site A, to 0.2–26.4 μM on site B (Paranhos et al., 2001) (**Table 1**). The same pattern is observed for fecal coliforms (**Table 1**). A State monitoring program (Environmental Institution of Rio de Janeiro State, INEA) shows that surface waters on the central channel and on the eastern part of the bay usually had fecal coliform counts below 1,000 MPN 100 ml^-1^ (INEA, 2013, 2014a^7^^,^^8^), which is the limit set by Brazilian law for recreational use (it should be pointed that this limit is higher than the one adopted in Europe and North America) (Conselho Nacional do Meio Ambiente, 1992). On the northern and northwestern parts, however, counts may reach values as high as 920 × 10^3^ MPN 100 ml^-1^ (INEA, 2013). From the data collected by INEA, it is possible to observe that the concentration of coliforms is also correlated with seasonal changes, increasing during the rainy season. Fecal coliforms counts in the area where aquatic sports of the 2016 Olympic Games will occur also extrapolate the limit of 1,000 MPN 100 ml^-1^ mainly from September to May (INEA, 2014a). Also, enterococcus values follow the same trend (INEA, 2014a). Furthermore, the uncontrolled growth of urban areas caused destruction of habitats and deforestation, which increased the runoff, silting up, and impairing even more water circulation in the western part of the bay. Remarkably, several areas that currently present better water quality are under the influence of the cities of Niterói and São Gonçalo, which are under steep population and industrial growth, and therefore under the risk of an increase in domestic and industrial load of wastewater. However, in these areas, there is the opportunity for concomitant implementation of sanitation, preventing the degradation that happened at the other parts of the bay, and which is much more difficult and expensive to restore.

The most concerning fact is that values for fecal coliforms (for the most contaminated areas in the western part of the bay) are above the limit throughout the year (INEA, 2013). This is a reflex of the poor water quality of the rivers on the basin. The water quality index (IQANSF, which takes into account: dissolved oxygen, biochemical oxygen demand, total P, NO_3_, pH, turbidity, total dissolved solids, temperature, and coliforms) determined by INEA during 2014 on the basin’s rivers for 55 stations, showed that, except for six stations for which data was not available, only five stations were classified as having medium IQANSF, all the others were classified as bad or very bad IQASNF^9^ (INEA, 2014b). Poor water quality of the basin’s rivers was also found by Aguiar et al. (2011), who studied four streams in the region of São Gonçalo (on the eastern part of the bay, usually found to be less polluted). They found that these streams were hypereutrophic, with abnormally high phosphate levels (from 4.35 to 130.8 μM, resulting in very low N/P ratios, and limiting primary production), and low oxygen values (from non-detectable to 3.72 ml l^-1^, presenting anoxia and hypoxia, respectively). The high input of nutrients into the bay results in strong hypoxic conditions near the bottom sediment (see OD values, **Table 1**).

Most studies that investigated long-term trends (e.g., Paranhos et al., 1993, 1995; Contador and Paranhos, 1996; Mayr, 1998) found an increase in pollutants load into the bay and a corresponding decrease in water quality. These studies detected increases on Chla *a* nitrogen, phosphorus, and coliforms, as well as a decrease in dissolved O_2_, concentrations, especially in the northwestern part of the bay, as consequence of an increase in urban areas without adequate sanitation. All these studies, however, point out that it is difficult to make conclusive analyses due to the irregularity of the data over time, because of the lack of a continuous and integrated monitoring program. A more recent study, which used a different approach to investigate water quality trends in Guabanabra Bay, casted new light on temporal trends. Borges et al. (2009) dated the P locked in sediment layers, showing that the bay is indeed being subjected to increasing eutrophication. They found three distinct periods, the first from 1810 to 1870s, a period of low level of P concentration [with total P (TP) concentration of 195 ± 23 μg/g, and a mean inorganic P (IP) of 47 ± 34%]; the second period up to the early 1900s, with a slight P increase (TP 2915 ± 30 μg/g, mean IP 63 ± 3%); and a third period, up to the present, of strong P enrichment (TP 1196 ± 355 μg/g, mean IP that 90 ± 3%). Besides the chronic load of pollutants into the bay, occasional acute loads can cause serious damages to the biota, as was the case of the rupture of an oil-pipe in 2000, which resulted in at least a 1,300 m^3^ of fuel spilled along the northern margin of Guanabara Bay. The spill reached the Guapimirim mangrove protection area, and affected marine life and the fisheries (Brito et al., 2009).

## Potentially Pathogenic and Harmful Microorganisms

It has been estimated a low-income population living around Guanabara Bay of ca. 4 million urban inhabitants, which corresponds to 45% of the basin’s population. This population lives in areas with no sanitation, i.e., their sewage is discharged untreated either into the bay or into its tributaries. This leads to serious environmental and human health problems: the rate of water-transmitted diseases is quite high (although sanitation has improved in Brazil, still circa 65% of hospitalizations are due to water-transmitted diseases) (Brasil, 2005). Microbes entering the bay also end up affecting other aquatic species and the ecosystem (Paranhos et al., 1995, 2001; Valentin et al., 1999; Guenther and Valentin, 2008; Gregoracci et al., 2012; Coutinho et al., 2014).

### Bacteria and Archaea

**FIGURE 4:**
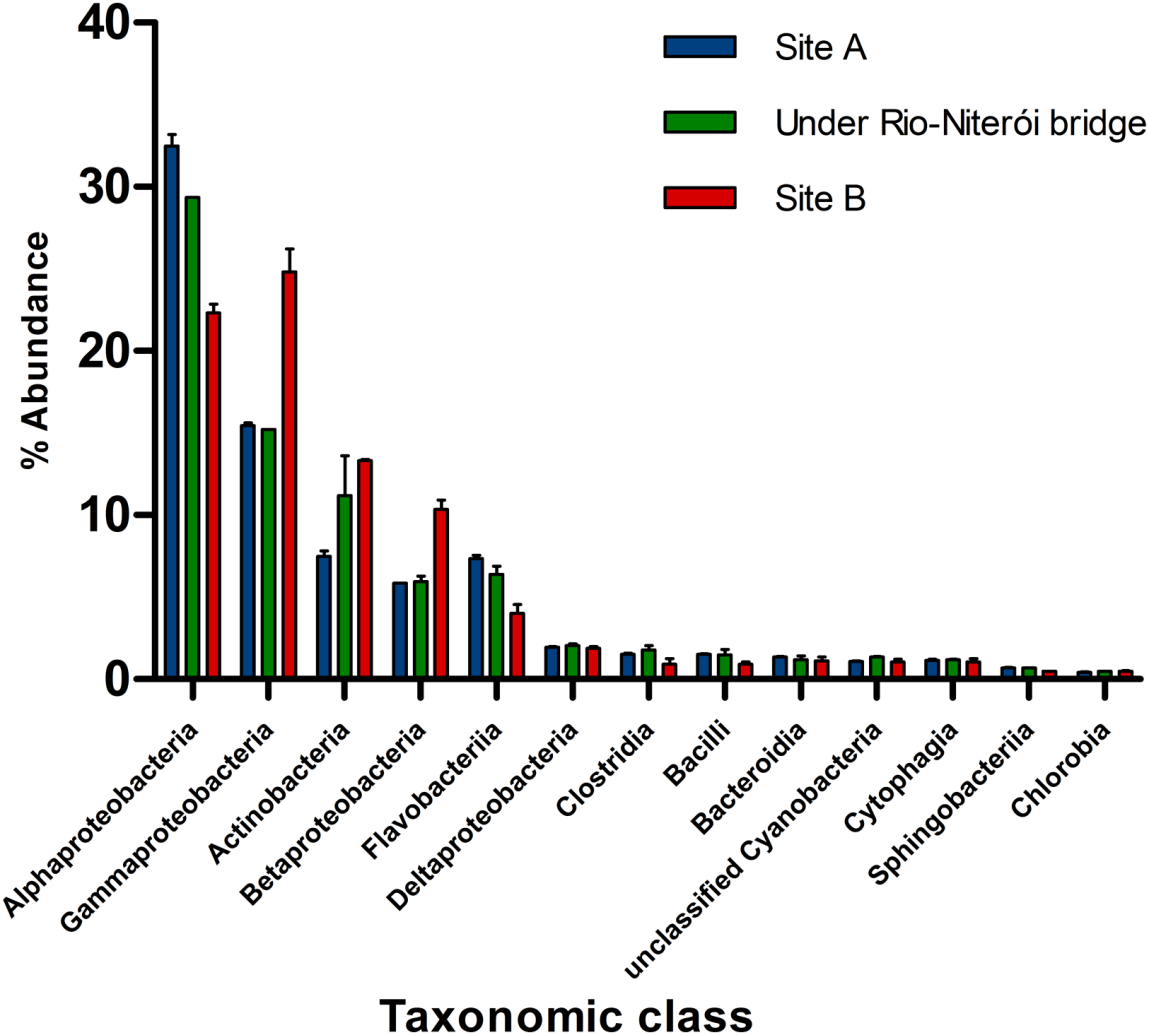
**Relative abundance of phylogenetic groups to metagenomes (at class level) in three sites of Guanabara Bay (modified from Gregoracci et al., 2012).** Location of sites are shown on **Figure 3**.

Recent metagenomic analyses of the Guanabara Bay water shed new light into how anthropogenic impacts modulate microbial communities (Gregoracci et al., 2012). Overall, Guanabara Bay is a heterotrophic system with lower abundance of microbial genes in the photosynthesis subsystem than in other tropical and temperate bays around the world based on metagenomics analysis (Gregoracci et al., 2012). Less impacted sites that are more influenced by the adjacent ocean, such as the central channel, are dominated by classes *Alphaproteobacteria* and *Flavobacteria*, which commonly dwell in oligotrophic waters. Meanwhile, highly impacted sites have microbial communities enriched by *Betaproteobacteria, Gammaproteobacteria*, and *Actinobacteria* (e.g., Alteromonadales, Pseudomonadales, Enterobacteriales, Oceanospirrilales, Chromatiales, Vibrionales, and Thiotrichales), which harbor several species that thrive in nutrient rich environments (**Figure 4**). Diversity levels inversely correlate with the degree of pollution (Vieira et al., 2008; Gregoracci et al., 2012). In the highly eutrophic sites, microbes metabolize organic matter at high rates, leading to depletion of dissolved oxygen, favoring anaerobes, and facultative anaerobes (e.g., *Firmicutes*) (Vieira et al., 2007, 2008). The pollution gradient is also reflected by the metabolic profile of the microbes. Despite the massive amounts of organic matter it receives daily, this environment is phosphorus limited (as evidenced by high N:P ratios). Guanabara Bay metagenomes are enriched with genes associated with phosphorus metabolism, when compared to other bays’ metagenomes. This may result from high abundances of phosphorus degrading organisms dwelling in this habitat, or reflect the necessity of this microbiome to optimize its phosphorus harvesting and utilization capacities (Gregoracci et al., 2012).

The highly impacted inner region shows the highest values of bacterial abundance and high nucleic acid content cells throughout the year. Both these variables were shown to be dependent on orthophosphate and ammonia concentrations. The inner portions of the bay are especially rich in methanogenic archaea (Vieira et al., 2007; Turque et al., 2010), a consequence of eutrophication, as methanogenesis occurs in anoxic areas with high concentrations of organic matter (Peng et al., 2008; Angel et al., 2011). Sponge associated archaea, as well as the diversity of ammonia oxidizing genes within them, were shown to adapt according to the gradient of anthropogenic impact to which the sponges are submitted (Turque et al., 2010). Thus, shifts in the microbial community due to eutrophication are likely to play an important role in the health and survivability of their eukaryotic hosts, which will also suffer the impacts brought by sewage contamination. Altogether, these results point to nutrient enrichment leading to a microbial community that has higher concentrations of microbial cells and a more active metabolism, promoting the dissemination of copiotrophic bacteria, such as vibrio. The number of culturable vibrios correlates positively with nutrient (e.g., orthophosphate and nitrite) (Gregoracci et al., 2012).

**FIGURE 5:**
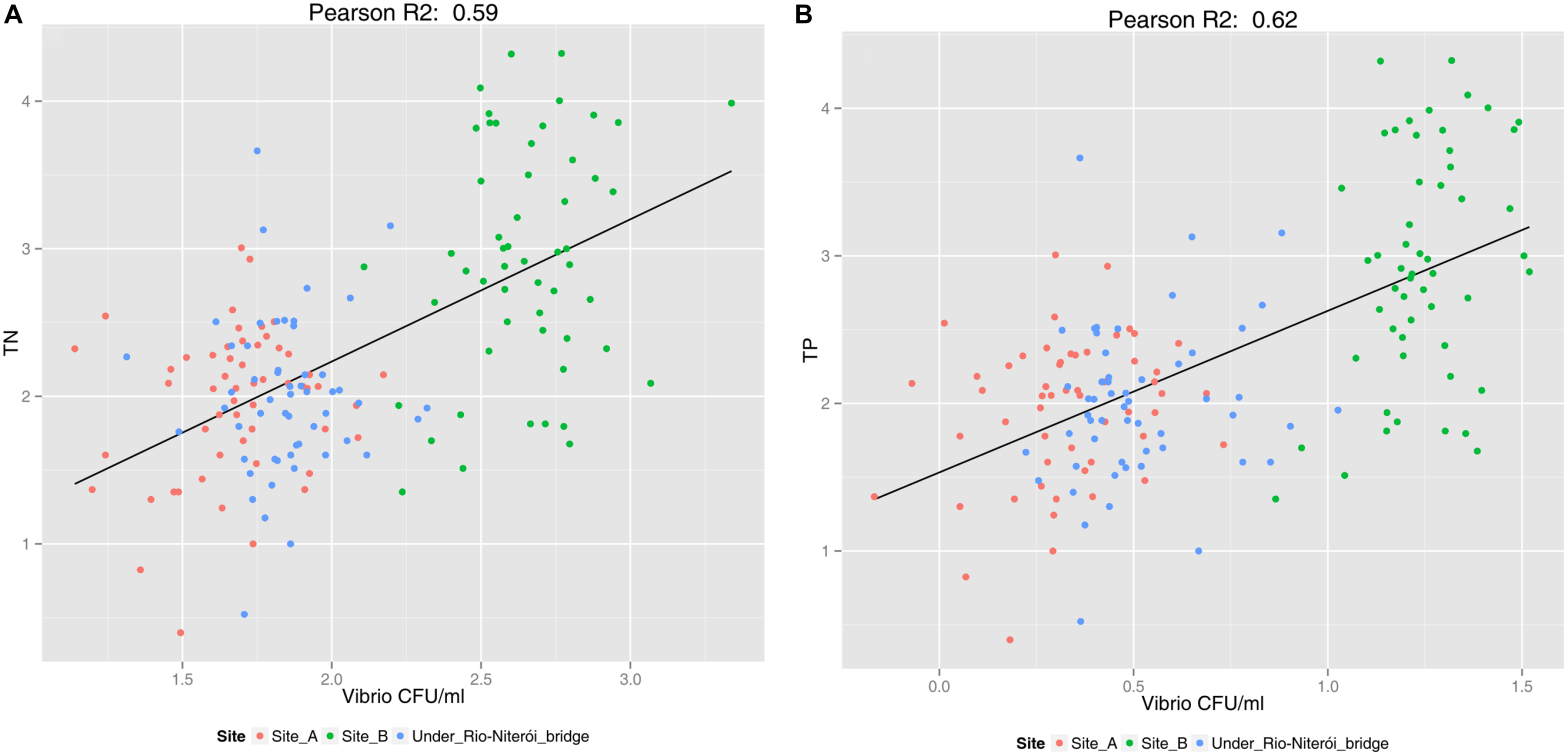
**Scatterplot showing the correlation between the abundance of vibrios (CFU ml^-1^) and concentrations of total nitrogen (log10 μM) (A), which had a correlation of *R*^2^ = 0.59, and total phosphorus (log10 μM) (B), which had a correlation of *R*^2^ = 0.62, for three sites of Guanabara Bay across a 6-years’ time-series (adapted from Gregoracci et al., 2012).** For site location see **Figure 3**. Besides the correlation between vibrio abundance and nutrient concentration, the figure shows a clear separation between the less impacted sites “A” and “under Rio-Niteroi Bridge”, and the more impacted site “B”.

Vibrios are indicators of water quality because they respond promptly and positively to nutrient enrichment (**Figure 5**) (Gregoracci et al., 2012). Gregoracci et al. (2012) found significant correlation between vibrio abundances and nitrogen and phosphorus concentrations (see **Figure 5**). There is, clearly, a higher abundance of vibrio on the more polluted “site B”, while the concentration of vibrios, though also correlated with nutrient concentration, is lower in the less polluted sites “A” and “under Rio-Niteroi Bridge” (**Figure 5**), which have a stronger influence of oceanic waters. Moreover vibrios are well known for harboring causative agents of a variety of zoonoses both to captive and wild aquatic animals, with risk of transmission to humans (Thompson et al., 2004). Pathogenic vibrio species, both to animals and humans, have been isolated from Guanabara Bay waters (Gregoracci et al., 2012.). Two pathogenic vibrio species previously isolated from Guanabara Bay (*Vibrio alginolyticus* and *V. parahaemolyticus*) and also the infective *Photobacterium damselae, V. harveyi*, *V. rotiferianus*, and *V. xuii* were retrieved from fishes (*Brevoortia aurea*, Clupeidae, known as Brazilian menhaden) during a mass mortality event in 2014. Mortality might have been multifactorial since toxic microalgae were also detected during the event, see section about microalgae). In any case, the presence of such pathogenic species are a clear threat to ecosystem and human health.

#### Opportunistic Pathogens and Drug Resistance

Water pollution favors species adapted to nutrient rich habitats, among which are included many potentially pathogenic bacterial lineages that are capable of infecting humans and other life forms (Smith and Schindler, 2009). Opportunistic pathogens can either thrive as free living or take up an infectious lifestyle, when in contact with a suitable host. Many genera that include potentially pathogenic bacteria were detected in Guanabara Bay, e.g., V*ibrio, Klebsiella, Pseudomonas, Clostridium*, and *Bacillus* (Vieira et al., 2008; Coutinho et al., 2014). When considering only the culturable fraction of Guanabara Bay’s microbiome, members of the orders Vibrionales and Clostridiales are extremely abundant, accounting for more than 50% of the antibiotic resistant culturable organisms (Coutinho et al., 2014). Exploring the diversity of potentially pathogenic organisms occurring at Guanabara Bay, and how it is affected by anthropogenic impacts, is fundamental to assess the potential risks to the local population.

The risk posed by these organisms is enhanced by increased resistance of some bacteria to antibiotics. The use and misuse of antibiotics throughout the decades has created a strong selective pressure that favors antibiotic resistant strains (Martínez, 2008). Sewage is one of the forms by which these organisms can spread from the human organism to the aquatic environment, and wastewater discharges have been shown to contribute to the dissemination of antibiotic resistant organisms and resistance genes (Kristiansson et al., 2011; Czekalski et al., 2012; Tacão et al., 2012). Highly impacted sites of Guanabara Bay (between Fundão Island and the mainland, and just north of Fundão Island, see **Figure 3**) were shown to be rich in antibiotic-resistant microorganisms. Several strains are classified as super-resistant, due to their capacity of tolerating drugs in doses up to 600 times higher than the clinical levels (Coutinho et al., 2014). Among these resistant and super-resistant lineages, several species of human pathogens were identified (e.g., *Vibrio cholerae, Klebsiella pneumonia*, and *Shigella* sp.), some of which are classified also as multi-resistant, capable of tolerating three or more different classes of antibiotics. The presence of resistant bacteria (KPC-producing bacteria) was recently reported in Guanabara Bay, making the media headlines (e.g., http://g1.globo.com). The occurrence of resistant bacteria was registered in two sites in a river that discharges into the bay, and in one site in the bay, at Flamengo Beach, which is located close to where the sailing Olympic competitions will occur. Taxa that are typical of the mammalian gut (e.g., *Enterobacteria, Firmicutes*, and *Bacteroidetes*) showing drug resistance were also identified. Altogether these facts indicate that untreated domestic and hospital waste/sewage are relevant sources of microbial contamination to the bay (Coutinho et al., 2014). Prevention of further dissemination of these organisms can only be achieved by providing adequate sewage treatment.

Contamination of Guanabara Bay’s waters by potential human pathogens has drawn attention from the international media, which has raised concern about the possibility of athletes participating in aquatic sports during the 2016 Olympic Games to fall ill because of the presence of the pathogens, especially viruses (Associated Press^10^; Denver Post^11^; NBC News^12^; The Guardian^13^). An independent investigation carried by the Associated Press revealed the presence of high levels of viruses (such as adenovirus, rotavirus, and enterovirus) and bacteria. Although the investigation was carried out by a researcher from the FEEVALE University, southern Brazil, the location, protocols, and exact data of the investigation are not available. There is no recent peer reviewed publication showing an investigation about virus presence in Guanabara Bay. The only report found dates back to 1975 (Homma et al., 1975). They investigated the presence of virus in several sites in the bay and found concentrations ranging from 19 to 1800 particles l^-1^, and transitory cytopathic effects on monkey kidney cells. They could identify the type of virus only for four samples, with all belong to the type ECHO virus (enteric cytopathic human orphan). The presence of pathogenic microorganisms in Guanabara Bay is undeniable. Among these microorganisms, the abundance, diversity and dominance of bacteria has been more thoroughly studied (Paranhos et al., 1998, 2001, Guenther and Valentin, 2008; Gregoracci et al., 2012; Coutinho et al., 2014). On the other hand, the lack of information about virus clearly indicates the necessity of more complete and systematic studies. Only with this kind of data it is possible to infer the treats to human health and determine efficient action plans.

### Microalgae and Harmful Algal Blooms

Environmental impacts in natural water bodies can be detected through changes in species composition, biomass, and in the trophic structure of natural communities. In these sense, aquatic microorganisms are reliable and efficient biosensors, as they have fast growth rates, and reflect environmental changes rapidly. Microalgae (O_2_-evolving photosynthetic protists and cyanobacteria) can be used to access environmental impacts through changes in Chla concentration (which is often used to indicate eutrophication, since levels of Chla present a good correlation with increase in nutrient loads), by the decrease in species number and diversity, and changes in species functional roles, e.g., shifts toward heterotrophy. Furthermore, some microalgae species may form harmful algal blooms (HABs), which cause impacts on the biota due to oxygen depletion or to toxin production. In the latter case, the effects can reach human populations by the consumption of contaminated seafood and inhalation of seawater aerosols (Hallegraeff, 1995).

Besides the pollution indicator parameters mentioned earlier, we also find high values of Chla in Guanabara Bay. Chla concentrations can be as high as 483 mg m^-3^ (Paranhos et al., 2001), indicating hypereutrophic conditions (Rast et al., 1989). During 2014, values reaching up to 700 mg m^-3^ were recorded during intense algal blooms in some locations. However, because Guanabara Bay is strongly influenced by tides, it is a dynamic environment that presents a large spatial and temporal variability. In general, mean Chla values across the bay detected during decadal monitoring programs ranged from 2.5 mg m^-3^ in the less polluted areas, to >100 mg m^-3^ in its inner parts (Mayr et al., 1989; Paranhos et al., 1998, 2001; Marques et al., 2004; Santos et al., 2007) (see **Table 1** and **Figure 3**). These values of Chla give a good representation of the spatial variability of the system, and demonstrate that they can be used as indicators of water quality. Values of primary productivity (PP) also show the system’s dynamics and influence of tides: PP ranged from 23 μgC l^-1^ h^-1^ during flood tide to 582 μgC l^-1^ h^-1^ during ebb tide (mean = 169 ± 152 μgC l^-1^ h^-1^) in the central channel ca. 7 km inward from the bay’s entrance (Guenther and Valentin, 2008). A “positive” effect of the eutrophication of the bay is that, because of its high PP, especially in the inner sites, coupled with the intense radiation and thermal stratification during summer, the bay plays a role as a sink of atmospheric CO_2_. The annual CO_2_ sink for the inner parts was calculated as -19.6 mol Cm^2^ yr^-1^, which matches the values of C found in the sediments) (Cotovicz et al., 2015).

The high values of Chla also highlight potential impacts on the aquatic community since they are usually connected to the dominance of one or few phytoplankton species (i.e., blooms of microalgae), and low species diversity. The consequences of lowered diversity are reflected on higher levels of the trophic web, especially if it is accompanied by changes in species functional role, and with cases of dominance by harmful microalgae species (Fistarol et al., 2003, 2004; Suikkanen et al., 2005; Granéli et al., 2008). Both these facts have been observed in Guanabara Bay. Heterotrophic/mixotrophic species have always been present in the bay. For example, about 25% of the dinoflagellates identified until 2010 were heterotrophic, several of them belonging to the genus *Protoperidinium* (Villac and Tenenbaum, 2010). However, based on the literature, it seems that there is an ongoing shift toward an increase in the frequency of dominance by heterotrophic organisms and toward smaller phytoplankton species. Valentin et al. (1999) reported the dominance of nanoplankton (flagellates and diatoms smaller than 20 μm) and filamentous cyanobacteria. Such dominance by nanoplankton was also reported by Santos et al. (2007), who found densities of 10^8^ cells l^-1^ of nanoplankton species, representing >57% of the phytoplankton community. Recent observations based on a monthly monitoring program (PELD Guanabara) carried out by our research group shows a somewhat permanent bloom of *Tetraselmis* spp. (Prasinophyceae). However, because of the lack of a long-term, uninterrupted monitoring in the bay, if this shift is occurring remains to be confirmed, as also remarked by Santos et al. (2007). The shift from an autotrophic to a heterotrophic community is an indication of water quality deterioration, and the presence of high loads of dissolved and particulate organic matter (Granéli et al., 1999). At the clearer waters at the entrance of the bay, phytoplankton community is dominated by autotrophs (except at depth, where the light decreases) (Guenther et al., 2012). On the other hand, Valentin et al. (1999) draw attention to the increase on phytoplankton biomass in the central channel, which may indicate a decreasing capacity of pollutants dilution by the incoming seawater. The pattern of shifting from phototrophic to smaller heterotrophic organisms linked to water quality deterioration was observed, e.g., in the Black Sea. Heterotrophic organisms may thrive on the increase in organic nutrients (Bodeanu and Ruta, 1998).

Some of the opportunistic microalgal species blooming in the bay may cause impacts on higher trophic levels due to the production of toxins or to oxygen depletion when blooms decline. The dinoflagellate *Scrippsiella trochoidea*, which has been reported in the bay since 1914, may cause fish kills due to anoxia, and is frequently found in densities as high as 10^6^ cell l^-1^ (Villac and Tenenbaum, 2010). Filamentous cyanobacteria (that may reach summer concentrations of 10^8^ l^-1^), and dinoflagellates from the genus *Prorocentrum* (Santos et al., 2007; Villac and Tenenbaum, 2010) are other potential toxin producers. The raphydophycean *Chattonella* spp. are fish-killing species that also have always caused problems in the bay, and it was detected by our group in concentrations of 3.54 ⋅ 10^6^ cells l^-1^ concomitantly to the aforementioned massive fish kill event that took place in October 2014. The presence of the domoic acid (neurotoxin) producing diatom *Pseudo-nitzschia* has also been detected, though in low abundances.

The relationship between increase in HABs and in eutrophication has concerned managers around the world, which try to implement measures to prevent factors that trigger these events (Anderson et al., 2002). As there are numerous examples from different regions around the world (e.g., Chesapeake Bay, U.S Coastal areas, Seto Inland Sea, Black Sea, Chinese coastal waters) showing a correlation between increase in eutrophication and frequency and magnitude of algal blooms, there are also many examples demonstrating that the regions that have implemented nutrient load controls have also witnessed reductions in phytoplankton biomass and harmful bloom events (Anderson et al., 2002). Some examples of regions where there was a decrease in HAB in connection to controlled reduction in nutrient inputs include Lake Washington, Seto Inland Sea, and the Black Sea (Edmondson, 1970; Bodeanu, 1993; Bodeanu and Ruta, 1998; Anderson et al., 2002; Imai et al., 2006; Nishikawa et al., 2014). High phytoplankton biomass cannot be sustained under low nutrient concentration, and there is consistent evidence showing that decrease in nutrient concentration will result in a decrease in biomass, and on the frequency of algal blooms. Edmondson (1970), Imai et al. (2006), and Davidson et al. (2014) showed significant correlation between decrease in Chl*a* concentrations and HABs, following actions to reduce nutrient concentrations in marine coastal ecosystems. These evidences demonstrate that high phytoplankton biomass and HABs are consequences of eutrophication that can be reversed if appropriate measures are taken to decrease nutrient inputs into water bodies. The presence of toxic and potentially harmful phytoplankton species in Guanabara Bay is a problem that may affect not only higher trophic levels of the aquatic food web, but also human populations. Thus, implementation of a continuous monitoring program is essential to provide information about the causes of these events. Such monitoring would allow the implementation of warning systems to the public during bloom events, and, following remediating strategies, to monitor the efficacy of the measures, besides giving important scientific information about the relationship of algal blooms and eutrophication.

Despite the polluted conditions of Guanabara Bay, microalgae richness is considerably high in its waters, with as many as 323 species described: 202 diatoms, 104 dinoflagellates, 9 cyanobacteria, 5 euglenophyceans, 1 chlorophycean, 1 prasinophycean, and 1 silicoflagellate (Villac and Tenenbaum, 2010). However, this high species richness does not reflect the diversity of the bay, since diversity is influenced by the relative abundance of species (i.e., if the environment is dominated by one or few species). Therefore, diversity is a better indicator of the impact level in the system than species richness. Nevertheless, the high number of microalgae species recorded demonstrates the great ecological relevance of this system and the importance of recovering it. The low diversity index found for the western margin of the bay (Shannon-Weaver index: 0.03-1.80 bits.cell^-1^, 50% < 1.00 bits.cell^-1^) indicates the instability of the system in that area, compared to the central channel that has a higher diversity index (Shannon-Weaner index: 1.30 -3.30 bits.cell^-1^, 35% > 1.00 bits.cell^-1^), and, consequently, a higher capacity to absorb disturbances (Villac and Tenenbaum, 2010).

## Consequences of Guanabara Bay Pollution to Higher Trophic Levels and to Human Populations

The impacted state of Guanabara Bay, especially at its most degraded areas, can be observed simply by looking at its shores and the color or transparence of the water. Additionally, severe effects of pollution can impact all levels of the aquatic trophic web. The microbial community of Guanabara Bay, for example, adapts to the pollution gradient, by altering its species composition and metabolic activities according to the local conditions. These changes, in turn, are reflected into all the upper trophic levels. Protozooplankton abundance is higher in the inner hypereutrophic parts of the bay (10^3^–10^5^ cell l^-1^) with dominance of small heterotrophic dinoflagellates and naked ciliates, while large marine dinoflagellates were only found at the entrance of the bay (Gomes et al., 2007). This confirms the trend of a shift from autotrophic to heterotrophic microorganisms in the bay. Paranhos et al. (2001) suggested that this higher abundance of protozooplankton in the inner sites could exert a top-down control on bacterial population in these areas. Zooplankton also responds promptly to pollution, and a decrease in copepods, appendicularia, cladocerans, and chaetognats numbers has been observed, concomitantly with the disappearance of siphonophores and thaliaceans in the most polluted areas (Valentin et al., 1999). Moreover, higher density of fish eggs and larvae were recorded in the less polluted areas at the entrance of the bay and in the central channel (Valentin et al., 1999).

A singular fact observed during early studies in Guanabara Bay is the different distribution of two dominant copepod species, *Acartia tonsa* and *Paracalanus parvus. P. parvus*, usually occurring in deeper and colder waters, almost disappeared from the inner part of the bay and does not seem to be adapted to highly polluted environments, whereas *A. tonsa*, found in estuarine waters worldwide, persists there in reasonably high proportions and appears capable of adapting and surviving in the unfavorable conditions of the polluted areas of the Guanabara Bay. Following Gomes et al. (2004) different ecological requirements of these two species rule their vertical migration behavior through the stratified water column at the entrance of the bay, with warm, low-salinity water from the inner bay at the surface and cold, high-salinity deep-ocean water below. *A. tonsa* maintains itself in the surface water layer during the night and does not seem to be affected by the presence of a sharp thermocline, whereas *P. parvus* shows a vertical migration limited to deeper waters, below the thermocline, a behavior that helps it to avoid being carried into the inner, polluted part of the bay (Gomes et al., 2004). Another important species of mesozooplankton is the cladocera *Penilia avirostris*. This species is an important component of the microbial loop between bacterioplankton and higher consumers because of its predation on bacterivorous microflagellates (Turner et al., 1988). This species decreases sharply in abundance from the entrance to the inner bay (Valentin et al., 1999). It is an interesting case of adaptation to the changing environmental conditions since this species has a complex reproductive strategy with the shift from asexual (parthenogenetic) to sexual (gamogenetic) phase under unfavorable environmental conditions, like the combined effects of pollution, low salinities and oxygen, leading to changes in trophic web structure (Valentin and Marazzo, 2003).

At the most degraded parts of the inner bay, the food web is highly compromised, fisheries yield have declined to 10% of the levels of three decades ago, mangrove areas have been reduced to 50% of their original size, and many of the beaches are not recommended for recreation (swiming) due to pollution (Marques et al., 2004; Coelho, 2007). Despite the accentuated degradation, there are still a number of families (around 6,000) that depend on the bay’s fisheries for their income (being sardines one of the most important catches), and also a number of people that practice recreational fishing (Marques et al., 2004). While fish stocks are stagnated (FAO, 2012), fishing effort has increased, producing a false idea of increase in fisheries yields (FIPERJ, 2011). Moreover, many species of cetaceans that used to be observed in the bay (e.g., *Balaenoptera edeni, Tursiops trucatus, Steno bredanensis*), are now absent. Only the Guiana dolphin (*Sotalia guianensis*) is still found in the bay, usually at the more clear waters of the central channel and around the mangrove’s protected areas (Bisi et al., 2013).

A clear example of impacts with consequences to human population was the aforementioned massive fish kill registered on October/November 2014. Although the fish species that died (*Brevoortia aurea*, Clupeidae, known as Brazilian menhaden) was not of significant commercial value, the intensity of the event, when at least 80 tons of dead fish were removed from the bay, caught attention of the media and raised concern from the government and the public. The causes of this particular massive fish kill could not be precisely determined, mostly due to the patchy nature of the phenomenon and lack of adequate monitoring previous to and during the event. Nevertheless, a thorough analysis of the microbiota, both free living in the water and associated to the fish, collected during the event point to some possible explanations. Firstly, in November, 2014, in one site where several fishes were found dead and moribund, we detected an algal bloom that turned the water yellowish and was composed almost exclusively by a gymnodinoid dinoflagellate of ca. 10 μm resembling the fish-killer genus *Karlodinium*, at densities higher than 60 × 10^6^ cells l^-1^. In another less intense fish kill event registered on February, 2015, once again an algal bloom was detected during our routine monitoring in the bay, formed by the ichtiotoxic raphidophyte *Chattonella* sp. in densities up to 3.54 × 10^6^ cells l^-1^. These two bloom-forming microalgal species are known fish-killer species in coastal waters worldwide (Imai and Yamaguchi, 2012; Place et al., 2012; Nishikawa et al., 2014). Their occurrence in the Guanabara Bay waters indicates a potential threat to fish stocks and a possible explanation to the recent fish kill events observed in the area. This is especially significant considering that the species that died was described to feed on phytoplankton, contrarily to most sardines that feed primarily on zooplankton (Sanchez, 1989). Besides potentially toxic microalgae in the water, several pathogenic vibrio species were isolated from the gills and kidney of moribund and dead fish we collected during the event. Sequencing of ca. 500 base pairs of the pyrH gene from approximately 50 bacterial isolates revealed the presence of *Vibrio harveyi*, *V. parahaemolyticus*, and *V. alginolyticus*, besides *Photobacterium damselae*. These are recognized pathogens of marine organisms, including fish, crustaceans and molluscs (Austin, 2010; Gauthier, 2015). Thus, two groups of microorganisms (toxic algae and pathogenic bacteria) with potential to harm and kill fish occur in the bay. Their role in massive fish-kill events (and also if we want to prevent future events) could only have been precisely assessed if a proper monitoring program was implemented. The duration and intensity of these fish-killing events was relatively new in Guanabara Bay. Thus, they should be taken as an alert and an opportunity to establish an adequate monitoring program. The presence of harmful algal species in Guanabara Bay had been registered (Santos et al., 2007; Villac and Tenenbaum, 2010), and the authorities are aware of that. However, it has not trigged yet a desirable response from them, as it has occurred in other coastal environments around the world that had this problem. In Seto Inland Sea, a region that played important role to the economic growth of Japan in the 1960’s, incidents of HABs had dramatically increased in connection to increase in eutrophication in the 1960s and 1970s, until a bloom of the toxic raphidophyte *Chattonella antiqua* in 1972 caused a large fish-killing event which resulted in large economic losses (7.1 billion yen). This bloom trigged the enactment of the “Law Concerning Special Measures for Conservation of the Environemnt of the Seto Inland Sea” and the “Total Pollutant Load Control” (TPLC). This prompt response from authorities decreased the nutrient load and, consequently, the number of HABs, which is maintained under control up to this date (Imai et al., 2006). The Seto Inland Sea case is a good example that, with proper regulation, economic and industrial development can occur without concomitant impact on the environment. Industrial production on that region continues to grow up to this date, but the TPLC guarantees that there is no increase in chemical oxygen demand (COD) on the water, consequently preventing the negative effects of eutrophication (Anderson et al., 2002; Imai et al., 2006).

## Current Actions for Remediation of the Bay

**FIGURE 6:**
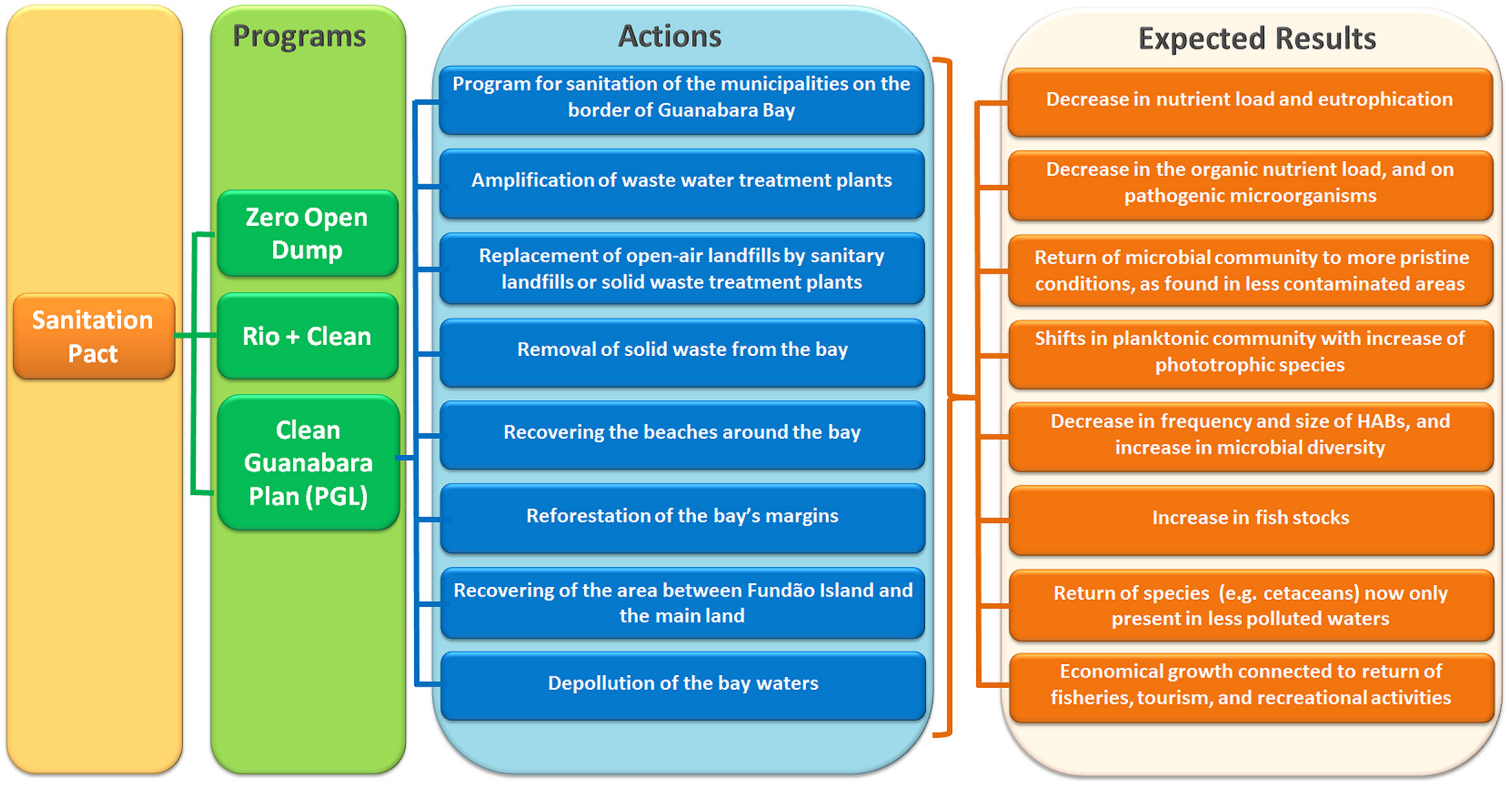
**Flowchart showing the three programs in which the Sanitation Pact, enacted by the state of Rio de Janeiro, is divided in.** From these three programs, the Clean Guanabara Plan (PGL) will tackle the pollution problems of Guanabara Bay, and it is divided in eight actions shown in the chart.

In 2011, the government of the State of Rio de Janeiro enacted the “Sanitation Pact”^14^ (Pacto pelo Saneamento), based on the Federal Law 11.445 (January, 2007) that established the national guidelines for sanitation. With this pact, the government intended to expand the access to sanitation for the population of Rio de Janeiro State, which encompassed (i) supply of potable water, (ii) wastewater treatment, (iii) urban cleaning services and solid waste management, and (iv) stormwater runoff drainage system. The pact is divided in three programs (**Figure 6**): (a) “Zero Open Dump” (Lixão Zero), which aims to replace open-air landfills by sanitary landfills or solid waste treatment plants; (b) “Rio+Clean” (Rio+Limpo), which aims to collect and treat 80% of the wastewater/sewage of Rio de Janeiro State until 2018; and (c) “Clean Guanabara Plan” (Plano Guanabara Limpa – PGL). This last one, PGL, aims to restore Guanabara Bay having as main goal to reduce in 80% the amount of sewage discharged into the bay. This goal was part of the commitments made before the International Olympic Committee (IOC) as part of Rio’s candidacy proposals as host for the 2016 games, which promised to properly treat 80% of the sewage entering the Guanabara Bay until 2016.

The PGL includes a series of actions (**Figure 6**), the main one being the “Program for Sanitation of the municipalities on the border of Guanabara Bay (Programa de Saneamento dos Municipios do Entorno da Baía de Guanabara – PSAM). PSAM received resources (1.5 billion Brazilian Reais, ca. 0.5 billion USD) from the Interamerican Development Bank (BID), and from the Government of Rio de Janeiro State, and intend to use 80% of this resource to implement sewage catchment systems and WWTPs on the municipalities around the bay, reducing the organic load discharged into the bay (SEA, 2011).^15^ Although it is not clear if all targets will be reached, some actions are being implemented, such as the expansion of the WWTP Alegria, which serves some of the regions with highest sewage load (such as Rio de Janeiro City Center, and the neighborhoods of Caju, Madureira, Maracanã, Grajaú, Vila Isabel, and others). The implementation of secondary treatment on Alegria WWTP already upgraded from 40 to 95% the reduction on the organic load of the treated sewage^16^ (Infraestrutura Urbana, 2011). The expectation for Rio de Janeiro State is to increase from the 2000 L s^-1^ (in 2006) to 13000 L s^-1^ of treated sewage (currently the state treats 4500 L s^-1^, which corresponds to a population of 2 million inhabitants). The total treated sewage already increased from 17 to 49%. To reach these marks, other WWTPs are also being expanded (e.g., WWTP Sarapui, and WWTP São Gonçalo) (Infraestrutura Urbana, 2011). However, the Government of Rio de Janeiro State already recognized that these targets may not be reached until 2016, and may demand more time for their conclusions (O Globo^17^).

The goals of the other actions of PGL follow the premises from the Sanitation Pact and include (**Figure 5**): (i) amplification of the WWTPs, (ii) replacement of open-air landfills by sanitary landfills or solid waste treatment plants, (iii) removal of solid waste from the bay, (iv) recovering the area between Fundão Island and the mainland, (v) recovering the beaches around the bay, (vi) reforestation of the bay’s margins, and (vii) depollution of the bay waters (this is the continuation of the program PDBG started in 1994 (see introduction), which did not reach its goals, but has now been reactivated). The Guanabara bay may also play an important recreational role. In the 1970s, several beaches in the inner bay (e.g., Ramos and Caju) were massively used for recreation, particularly by the population living in the north area of Rio de Janeiro city.

Microorganism can be studied to assess the effectiveness of the clean-up strategies. If the targets of the PGL are reached and the WWTP are upgrade and new ones are built, the input of organic nutrient into the bay will decrease considerably. Consequently, a gradual change in the microbial community toward: (i) an increase in *Alphaproteobacteria* and *Flavobacteria* is foreseen, with also (ii) a shift back to phototrophic dominated phytoplankton community (as previous conditions, and as it was observed in the Black Sea, see Bodeanu, 1993), and (iii) a decrease in Chla concentration and on the frequency of algal bloom (Edmondson, 1970; Anderson et al., 2002; Imai et al., 2006). A decrease in the frequency of algal blooms, including HABs, is directly correlated to a decrease in nutrient concentration (mostly P in freshwater environments, and N in marine and estuarine waters) (Anderson et al., 2002), as demonstrated by Nishikawa et al. (2014), Imai et al. (2006), and by Edmondson (1970). Therefore, the completion of the WWTP and the other measures planned to remediate the current conditions of Guanabara Bay would produce a chain of positive results by reducing the nutrient load, the input of pathogens, including human, and decreasing the algal bloom and promoting phytoplankton diversity, which would have effects on the whole trophic web. Remediating strategies applied in other regions (e.g., Chesapeake Bay, Seto Inland Sea, Sydney Harbor) demonstrate the importance of a continuous monitoring program, which allows following the efficacy of the measures applied. Furthermore, it represents an excellent opportunity to study long-term shifts in the microbial community. Although remediating plans are costly, they bring undeniable benefits to the society, from decreasing health risks to providing clean recreational site, and increasing landscape’s aesthetical value. In addition, it can generate monetary benefits such as increase in fisheries, tourism, recreational activities, and, consequently, economic sectors that provide services to supply/maintain these activities. It also increases the value of waterfront land. Furthermore, when society perceives water quality improvement, they increase their willingness to pay for its improvement, as it was observed in Chesapeake Bay (Bockstael et al., 1989; Leggett and Bockstael, 2000).

## Perspectives

Pollution in Guanabara Bay reflects the neighboring social conditions of this vast and densely populated hydrographic basin. If we are to recover the bay, besides remediating environmental degradation and treating contamination sources, it is important to implement social projects that address the causes of the problem. This is one of the most densely populated areas in the world, which includes numerous slums that do not have basic sanitation systems. The projects being implemented for remediation of the bay, tackle exactly these points, however, remediation plans on this scale take years to produce the expected effects, and should not, therefore, be dependent on political mandates. Even if these projects are implemented by federal or state government agencies, they would need to be carried and followed by academic research institutions, which are independent, and not limited by mandate’s time, and also organizations representing the population, which are the main stakeholders interested in the recovery of the bay. The experiences from the recovery of other bays around the world should be used as examples of what works and what is necessary for an efficient recovery strategy. Chesapeake Bay, for example, has been carrying a program to recover the watershed’s water-quality for nearly three decades^18^^,^^19^ (the Chesapeake Bay Program – CBP). The success of the program is based on some specific characteristics: (i) the involvement of all states encompassing the watershed, establishing a total maximum daily load (TMDL) of nutrients into the watershed (which is identified by a watershed implementation plan); (ii) setting 2-years milestones (which allows quick rectifications of plan); (iii) implementing a track and assess progress system, and (iv) federal intervention (Federal Actions) if milestones are not achieved. An important strategy used to guarantee the success of the program is the transparence of the results. Therefore, part of the resources of the program are allocated to provide mechanism to inform the population of the progress, such as the Chesapeake Tracking and Accounting System (BayTAS), an interactive on-line tool that the public uses to track progress of the implementation of TMDL. Only if the stakeholders feel involved and see the results, they will contribute to the recovery. The experience from the recovery of other coastal areas around the world also reveal that remediation programs are not an easy task and some of the results may take time to produce the desired effects. Sydney Harbor, in Nova Scotia, was one of Canada’s most contaminated sites. In 2004 the government of Canada and Nova Scotia committed to remediate the area, which began in 2009. The remediating program was followed by a monitoring program to check the recovery of the system. This monitoring revealed that, mostly, the remediation program reduced the concentration of contaminates (e.g., of polycyclic aromatic hydrocarbons – PAHs, and, to some extent, polychlorinated biphenyls – PCBs); however, metals (As, Cd, Cu, Hg, Pb, Zn) showed little spatial-temporal variability (Walker et al., 2013a,b; Walker and MacAskill, 2014). In the Guanabara Bay water circulation is high, pointing out to an optimistic scenario in face of environmental sanitation.

Monitoring programs provide data and scientific publications for informed decision-making, for evaluation of water quality conditions, and to build predictive models (which are used for implementation of management strategies). Monitoring programs need to be implemented and supported by the State. A good example of long-term monitoring is the one carried in the Seto Inland Sea by the Fisheries Technology Institute of Hyogo Prefecture (Imai et al., 2006; Nishikawa et al., 2014). A monthly monitoring sampling is made since 1973, and the data collected allowed to make, for example, correlations between the phytoplankton abundance and nutrient levels, and then, to predict the occurrence of algal blooms based on the nutrient availability (Imai et al., 2006; Nishikawa et al., 2014). To ensure its continuity, the monitoring is carried by a government-funded institute, and the data is publicly available.

## Conclusion

Guanabara Bay has an environmental, social, and economic importance for the region around its basin. Its current state endangers wildlife and poses risks to human populations that use this water resource. As highlighted in this review, Guanabara Bay waters harbors opportunistic pathogenic microbes capable of harming humans and several other life forms. The population of Rio de Janeiro is often in contact with the bay’s water, be it directly (e.g., by bathing in its waters or in the nearby oceanic beaches) or indirectly (e.g., through the consumption of seafood). Thus, the restoration of Guanabara Bay is not only of ecological, social-cultural and esthetic relevance, but is also a public health issue. Despite enduring decades of severe environmental degradation, Guanabara Bay still present some resilience and, because of its hydrodynamic characteristics, the capacity to recover from these impacts, providing that sanitary measures to recover the bay and prevent further degradation are taken. The current sanitary conditions of the bay are not worse than those of other heavily contaminated bays around the world before they had been successfully recovered. It has been assumed that it may be possible to restore the bay’s water quality if ca. 80% of all domestic and industrial sewage are appropriately treated. Therefore, a remediating plan implemented by the government, with the participation of all stakeholders, may produce the desired effects in medium and long run.

An important feature of the bay, provided by the researches that have been carried in the bay, is its heterogeneity, with the inner parts having lower circulation, higher contaminates discharge, and consequently, worse water quality. A continuous monitoring program will be needed to evaluate the restoration plan of Guanabara bay, and the results should be available to the population. If all ongoing plans are implemented, the restoration of Guanabara Bay and its shores may be one of the best legacies of the Olympic Games in Rio de Janeiro.

## Author Contributions

GF, CT, PS, and FT conceived and designed the article. AC, SP Jr. RV made all the maps presented in the article. GF wrote the manuscript. FC, AM, wrote the section “Bacteria and Archea” and “Opportunistic Pathogens and Drug Resistance” with contributions of CT, FT, TV, and RK. PS and DT contributed to the writing of the Microalgae and Harmful Algal Blooms section. RP, PS, RC, DT, RP, CR contributed to the section “Water Quality: pollutants in the Bay”. JV,RM, and GF contributed to the writing the section “Consequences of Guanabara Bay Pollution to higher Trophic Levels and to Human Populations”. All authors provided scientific expertise and all authors contributed to the editing of the manuscript.

## Conflict of Interest Statement

The authors declare that the research was conducted in the absence of any commercial or financial relationships that could be construed as a potential conflict of interest.

## Footnotes

1 http://www.demographia.com/db-worldua.pdf

2 www.ibge.gov.br

3 https://sustainabledevelopment.un.org/content/documents/Agenda21.pdf

4 http://www.comitebaiadeguanabara.org.br/sig-rhbg/

5 http://g1.globo.com/natureza/blog/nova-etica-social/post/estudo-feito-por-fundacao-alema-mostra-que-baia-da-guanabara-recebe-18-mil-litros-de-esgoto-por-segundo.html

6 http://oglobo.globo.com/esportes/pezao-admite-que-sera-dificil-alcancar-os-80-de-despoluicao-da-baia-ate-os-jogos-de-2016-15415948

7 http://download.rj.gov.br/documentos/10112/2026700/DLFE-68424.pdf/GraficoQualidadedasAguasdaBaiadeGuanabara.pdf

8 http://www.inea.rj.gov.br/cs/groups/public/documents/document/zwew/mdq3/~edisp/inea0047160.pdf

9 http://www.inea.rj.gov.br/cs/groups/public/documents/document/zwew/mdi2/~edisp/inea0026989.pdf

10 http://bigstory.ap.org/article/d92f6af5121f49d982601a657d745e95/ap-investigation-rios-olympic-water-rife-sewage-virus

11 http://www.denverpost.com/sports/ci_28558663/ap-investigation-dirty-rio-water-threat-at-2016?source~=~skipframe-feedspot.com

12 http://www.nbcnews.com/health/health-care/dirty-water-blamed-sick-rowers-brazil-olympic-trial-n407456

13 http://www.theguardian.com/sport/2015/jul/31/alistair-brownlee-calm-despite-rio-2016-pollution-fears-at-olympics

14 http://www.rj.gov.br/web/sea/exibeconteudo?article-id=330838

15 http://www.rj.gov.br/web/sea/exibeconteudo?article-id~=~1055505

16 http://infraestruturaurbana.pini.com.br/solucoes-tecnicas/4/artigo220154-2.aspx

17 http://oglobo.globo.com/rio/coi-diz-esperar-que-compromisso-de-despoluir-baia-de-guanabara-seja-cumprido-ate-2016-15438299

18 http://www.epa.gov/reg3wapd/pdf/pdf_chesbay/TMDLPoster7-13.pdf

19 http://www2.epa.gov/chesapeake-bay-tmdl
